# Recent Advances in Biomaterials for 3D Printing and Tissue Engineering

**DOI:** 10.3390/jfb9010022

**Published:** 2018-03-01

**Authors:** Udayabhanu Jammalamadaka, Karthik Tappa

**Affiliations:** Mallinckrodt Institute of Radiology, School of Medicine, Washington University, Saint Louis, MO 63110, USA; kktappa@wustl.edu

**Keywords:** three-dimensional printing, additive manufacturing, bioprinting, biomaterials, bioinks, ceramics, polymers, composites, tissue engineering

## Abstract

Three-dimensional printing has significant potential as a fabrication method in creating scaffolds for tissue engineering. The applications of 3D printing in the field of regenerative medicine and tissue engineering are limited by the variety of biomaterials that can be used in this technology. Many researchers have developed novel biomaterials and compositions to enable their use in 3D printing methods. The advantages of fabricating scaffolds using 3D printing are numerous, including the ability to create complex geometries, porosities, co-culture of multiple cells, and incorporate growth factors. In this review, recently-developed biomaterials for different tissues are discussed. Biomaterials used in 3D printing are categorized into ceramics, polymers, and composites. Due to the nature of 3D printing methods, most of the ceramics are combined with polymers to enhance their printability. Polymer-based biomaterials are 3D printed mostly using extrusion-based printing and have a broader range of applications in regenerative medicine. The goal of tissue engineering is to fabricate functional and viable organs and, to achieve this, multiple biomaterials and fabrication methods need to be researched.

## 1. Introduction

The human body has incredible capacity to regenerate, but this regeneration is limited by factors such as the type of tissue, and the need for growth hormones for differentiation and physical size (critical defect). Any injury to a tissue beyond this critical size needs external support. This approach of supporting tissue regeneration is often referred to as tissue engineering (TE) or regenerative medicine (RM). The external supports are called scaffolds. These scaffolds create a platform for the cells to migrate to the site of action and forms new tissue. Hence, scaffolds play an important role in TE and regenerative medicine. These scaffolds are often loaded with growth factors to hasten differentiation of cells to preferred types of lineage to promote new tissue formation. The physical and chemical composition of scaffolds is critical for cell viability and cell proliferation. 

There are two critical factors that shape the use of scaffolds: the choice of biomaterial to create a scaffold and the method of fabrication. Much research has been done on modifying and creating new biomaterials. Biomaterials are defined as any materials that interface with biological systems. Biomaterials are classified based on many criteria such as chemical and physical composition, biodegradability, type of origin, and generations of modifications [1,2]. Depending on the target tissue, the choice of biomaterial is made. In recent years, much focus was towards engineering biodegradable biomaterials. Based on the chemical composition, biomaterials are classified into ceramics, polymers, and composites. The ceramics class of biomaterials have major components of inorganic metal compounds and/or calcium salts. These biomaterials have been primarily used in orthodontal applications. Polymers are used in soft TE because of their similarity with connective tissues. The composite class of biomaterials are blends of ceramics and polymers. These composites have applications in orthopedic and dental TE.

Polymers of natural and synthetic origin are widely used in TE and RM. Naturally-occurring biomaterials, such as collagen, chitosan, hyaluronic acid, alginate, etc., are widely used because of their biodegradability, biocompatibility, and abundant availability. Degradation of biomaterials is one of the important features for naturally-available polymers. As these biomaterials are present in extracellular matrix (ECM), cells have good compatibility and growth response. Collagen is one of the most widely-used naturally-occurring biomaterials in scaffolds in various applications. The ubiquitous nature of these proteins allows their usage across different species without immunogenic reactions. Several commercially-available collagen-based scaffold materials are available under different brand names, such as Zyplast, Zyderm, Collagen Meniscal Implant, Contigen, etc. A second commonly-used naturally-occurring scaffolding material is chitosan. The chemical structure of chitosan is similar to another ECM molecule, hyaluronic acid. The rate of degradation of this biomaterial can be controlled by the degree of acetylation. A recent approach to creating scaffolds with naturally-occurring biomaterials is intact ECM. Organs/tissues from different species are prepared by decellularization. In this method, the whole ECM stays intact while all the cellular components are removed. These scaffolds from xenogenic origin were clinically used in many human subjects. Other naturally-occurring biomaterials that are used in RM and TE include alginate, gelatin, agarose, and hyaluronic acid [3].

Synthetic biomaterials are either modified naturally-occurring biomaterials or completely synthetic. These biomaterials offer choices of degradable and non-degradable biomaterials. Non-degradable synthetic biomaterials, such as polyethylene derivatives, poly(tetrafluoroethylene), poly(methyl)acrylates, polyacrylamides, polyethers, polysiloxanes, and polyurethanes are widely used. These non-degradable biomaterials have advantages such as non-immunogenic, reproducible quality, and tailored mechanical properties and shapes. Many orthopedic implants, sutures, catheters, and fracture-fixing devices are manufactured using these non-degradable biomaterials. The class of degradable biomaterials include polyesters, poly(α-hydroxy acids), polylactones, polyorthoesters, polycarbonates, polyanhydrides, and polyphosphazenes. These synthetic biomaterials are used not only as scaffolds, but also as drug delivery systems. Thus, these biomaterials can be tailored to defined degradation kinetics along with desired growth factor release rates [4].

In this review, 3D printing as an approach to fabricate scaffolds and organs is discussed. Among different types of 3D printing techniques, extrusion-based and inkjet-based 3D printing methods are commonly used for bioprinting. Two types of constructs are 3D printed for TE and RM: acellular scaffolds which contain biological components, and cell-laden scaffolds for tissue mimicry [5]. Biomimicry, autonomous self-assembly, and mini-tissue building blocks are the three major approaches used for 3D bioprinting. In biomimicry type of approach, cellular components are arranged/reproduced to mimic a living tissue whereas, in autonomous self-assembly, early cellular embryonic components are used to produce their own ECM, cell signaling, and cellular architecture. In the mini-tissue fabrication approach, the smallest structural and functional units of a tissue are assembled into a larger tissue/organ. For fabricating a complex multifunctional tissue, a combination of all these three approaches is required [6].

In the inkjet printing method, biological materials are selectively placed onto the build platform in a layer-by-layer manner until the required construct is formed. The droplets are formed either by piezoelectric or thermal actuation. In piezoelectric droplet formation, voltage pulses induce the pressure change resulting in droplet formation whereas, in thermal actuation, a heating element vaporizes the biomaterial and deposits a droplet. Inkjet printers are known for their high speed, precision, and wider biomaterial availability. Due to the high controllability of droplet size and deposition rate these printers have the ability to print constructs with high resolution and accuracy [7].

In extrusion-based bioprinting, biomaterials are extruded from the print-head due to the exertion of mechanical or pneumatic pressure. This technique does not involve heating processes and, thus, enables convenient incorporation of cells and bioactive agents. Compared to inkjet 3D printing, extrusion-based 3D printing enables a continuous flow of the biomaterials resulting in an ease of operation and wider selection of biomaterials, including polymers, decellularized matrices, cell-laden hydrogels, spheroids, and aggregates [8].

Modern 3D printing machines allow fabricating complex multicellular tissue/organ due to their ability to use multiple print heads loaded with different cell lines. Cui et al. used a dual 3D bioprinting technique to fabricate large functional bone grafts with organized vascular networks [9]. Want et al. used a novel building-block approach to 3D print complex organ-regenerative scaffolds, such as liver tissue [10]. Similarly, many researchers have used different approaches with varied biomaterials to fabricate living tissues/organs, including ovaries [11], skin [12], aortic valves [13], and bone and cartilage [14]. Although, many living tissues were successfully fabricated in the lab as a proof of principle, a fully-functional life-size human organ fabrication is still in its infancy.

## 2. Need for Scaffolds and Tissue Engineering

Tissue engineering offers an alternative method to address the issue of ever increasing need for organ transplants. Data from the Organ Procurement and Transplant Network (OPTN), indicates that as of January 2018, over 115,000 patients needed organ transplant, while only 34,769 transplants were performed [15]. Using approaches from TE and RM, the gap between the number of patients awaiting transplants and donors available can be filled. In degenerative diseases affecting organs, such as the kidneys, liver, pancreas, and heart, the organs fail completely and organ transplant from another human is the only available treatment modality. Patients on a waiting list may or may not have the time to wait until they receive an organ donation, leading to death of 20 patients every day [16]. The aim of TE is to create functional organs from patients’ own cells. This process is not a simple task as it involves multiple factors of human physiology, such as culturing multiple cell types, vasculature, nerve innervation, and interactions with surrounding tissues. These challenges are being researched and new strategies are being developed to overcome these factors.

**Figure 1:**
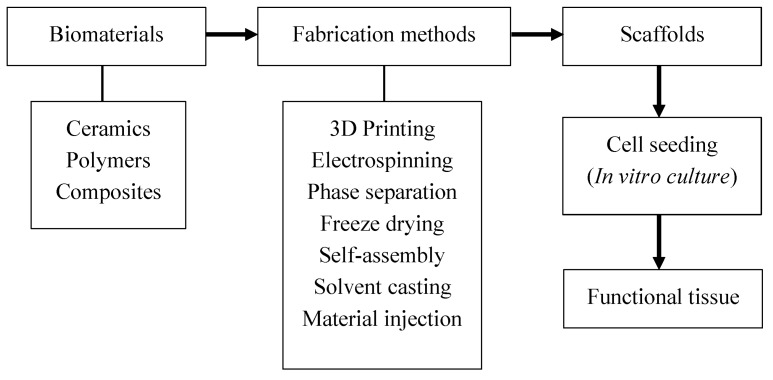
Flowchart for creating functional tissues from biomaterials.

The process of TE starts with biomaterials followed by the fabrication of scaffolds. These scaffolds are chemically and physically modified during the fabrication process to meet specific needs, such as biodegradability, porosity, size, shape, and bioactivity. These requirements may vary depending on the nature of the biomaterials, the fabrication process, and the target tissue. After making the scaffold with the desired properties, the scaffold can be seeded with cells and cultured in vitro to create the desired tissue, or can be placed within the body and have the host cells infiltrate the scaffold and populate. Growth factors, hormones, and chemical cues are key in both these approaches as they define cell differentiation and functionality of the cultured tissue. This process is shown as a flowchart in Figure 1.

The main constraint for biomaterial usage in TE and RM is its ability to mimic the ECM that support cell viability and cell functionality. Characteristics of biomaterials that need to be considered are surface chemistry, surface reactivity, surface roughness, surface charge, contact angle, and rigidity [17]. These properties, in turn, determine the cell-biomaterial interactions and cell-cell interactions. These cell interactions are key to cell attachment, viability, and differentiation, which determine the success of the scaffold. Few of the important physicochemical prerequisites for biomaterials include supporting cell survival, inducing differentiation, promoting cell adhesion, stimulating cell response, the ability to deliver therapeutics, biocompatibility, biodegradability, adaptability in the fabrication process, mechanical strength, directional stability, and sterializability [18]. Biomaterials not only provide for physical support for cell attachment, but also to deliver therapeutic agents, such as drugs, proteins, growth factors, and chemical cues. Most of the mammalian cells are anchorage-dependent for viability. The absence of a substrate for cell attachment often results in cell death. Hence, the surface chemistry and structure of scaffolding materials are of high importance for cell viability and function.

Biomaterials’ and scaffolds’ surfaces are modified to promote cell adhesion using three strategies—chemical modification, physical modification, and surface coatings. Several methods are used to modify the biomaterial’s surface chemistry using radiation and chemical reactions. These chemical modification methods include UV irradiation, oxidation, acetylation, alkali hydrolysis, silanization, and the addition of glycidyl groups. The addition of arginine-glycine-aspartic acid (RGD) groups on the surface of biomaterials is an established method to promote cell adhesion. These chemical modifications result in the addition of several functional groups that promote cell adhesion, proliferation, and gene expression. Physical modifications imply structural changes on the surface or bulk of the biomaterials. Using techniques such as sandblasting, plasma etching/spraying, mechanical polishing, and photolithography, surface roughness and topography were engineered [19]. Surface coating of scaffolds is achieved using several methods, such as solvent casting, vapor deposition, Langmuir-Blodgett deposition, surface grafting, sol-gel coating, electrophoretic deposition, precipitation, thermal treatment, steam treatment, and dipping methods. These coatings can change multiple surface properties of the scaffolds leading to better cell-biomaterial interactions.

The goal of scaffolds and biomaterials is to support cell proliferation and function. This leads to the next step of introducing cells onto the scaffolds. Conventional approaches for the addition of cells to scaffolds include seeding scaffolds with the desired cells. Cell suspension in growth media is added onto the scaffold surface and cell migration into the scaffold bulk is expected. The source of cells to be seeded depends on the target tissue. Stem cells and pluripotent stem cells are most widely used because of their ability to differentiate into specific cell lines depending on the stimulus. Ideally, these cells from the host need to be cultured to fabricate organs with zero immunogenic reactions. Bone marrow stromal cells (BMSCs), mesenchymal stromal cells (MSCs), and stem cells from amniotic fluid or placenta are good prospective cells for organ fabrication. In the fabrication of complex organs, consideration is given to the primary cells that perform the specific function of the organ, along with supportive cells that are involved in the secretion of the supportive matrix, vasculature, and structural framework. To fabricate such organs, primary cells of different genotypes and phenotypes can be added, or pluripotent cells can be added that differentiate into the required cell lines [20].

## 3. Scaffold Fabrication Methods

There are several methods for scaffold fabrication. The fabrication process is the step after which the biomaterials are transformed into scaffolds. These fabrication methods are physical and/or chemical processes that are performed on biomaterials to render them usable for tissue engineering. Not all biomaterials are suitable for a given fabrication method. Hence, biomaterials are constantly modified to enable their use in each fabrication method. Conventional fabrication methods include electrospinning, phase separation, freeze drying, self-assembly, solvent casting, textile technologies, material injections, and additive manufacturing [21,22]. Each fabrication method has its own advantages and disadvantages. To overcome certain disadvantages, these fabrication methods may be used in combination with other methods.

In electrospinning, an ionic solution of polymer is ejected through a fine orifice across a high voltage potential. Due to the potential difference, the polymer solution is sprayed as fine fibers while the solvent vaporizes, leaving a polymeric scaffold. Although a large variety of polymers can be used in this process, fabricating scaffolds with complex geometries and structures is still a limitation. Highly porous and complex three-dimensional scaffolds can be fabricated using phase separation method. In this method of scaffold fabrication, a solution with different solvent systems is used. Using thermal or non-solvents, one of the phases is separated leaving only the desired polymer solution. Using this method, the porosity of scaffolds can be controlled, but is limited by polymer variety and inability to fabricate high-resolution scaffolds. Freeze drying uses the principle of sublimation to remove water or solvent from a system to yield porous scaffold. This method also suffers similar limitations as phase separation. The self-assembly method of fabrication relies on ionic interactions among polymers to organize themselves to form a scaffold. Scaffolds formed using this method have very poor mechanical properties for three-dimensional scaffolds. Additionally, the expensive costs for fabrication of these scaffolds limits their use in RM. In textile-based fabrication methods, fibers form polymers and composites are knitted, woven, and braided to create scaffolds. This fabrication method has many advantages, including the ability to create porous, pre-vascularized, and permeable scaffolds [23]. Disadvantages for this scaffold fabrication method are its inability to encapsulate within the scaffolds and the harsh manufacturing process [24].

Additive manufacturing, commonly referred to as 3D printing, includes stereolithography, inkjet printing, bioprinting, fused deposition modeling (FDM), extrusion, laser beam melting, selective laser sintering (SLS), digital laser printing (DLP), electron beam melting, and polyjet [25,26]. Irrespective of the technology used for 3D printing, all the methods in additive manufacturing use the same principle of laying down materials in a layer-by-layer fashion until the whole object is created. In other words, a 3D object is built by successive addition of 2D layers of material. Additive manufacturing was initially used in mechanical and industrial applications for creating prototypes later adapted by various industries. This method of fabrication has several advantages, including the ability to create complex geometries, multiple materials, and a wide range of biomaterials can be used. Using biodegradable biomaterials and cells, researchers have developed novel strategies and methods to create tissues with multiple cell lines and organs [26,27,28].

Using patient data from CT/MRI, these scaffolds can be designed specific to that patient. 3D models of defective regions of tissues can be identified and 3D models can be prepared [29]. Using advanced computer aided designing (CAD) software, porosity and structures for vasculature can be added to these models. Using the advantage of working with multiple cells and biomaterials in 3D printing, functional organs and tissues can be fabricated. Due to the ease and ready availability of these resources, 3D printing is gaining significant importance as a fabrication method for TE and RM.

## 4. Properties of Biomaterials That Make Them Suitable for 3D Printing

The principle of bioprinting is that the biomaterial, in the form of liquid, is printed layer by layer method until the whole object is fabricated. Immediately after the biomaterial in liquid form leaves the print head, the biomaterial is solidified to retain the shape. This process of converting from sol to gel or phase transition process is the key for a biomaterial to be adapted in bioprinting. Polymers and composites are most widely used because they can be polymerized using various methods, rendering them “3D-printable”. Factors that are important to make biomaterials suitable for 3D printing processes are rheological properties and the method of crosslinking. These properties are, again, dependent on the method of bioprinting, i.e., requirements for bioinks used in inkjet printing are different from extrusion-based bioprinting. Additionally, suitable properties for printing depend on the nature of constituents in the given polymeric biomaterial. Bioinks containing live cells need consideration on the shear forces that act on cells during the printing process along with other rheological properties.

Rheological properties, such as viscosity, non-Newtonian behavior, Barus effect, and the method of crosslinking are to be considered while designing novel polymeric or hydrogel systems for bioprinting. Shear thinning non-newtonian fluids are ideal as they exhibit low viscosity when subjected to shear forces and are not time-dependent. Polymers are subjected to shear and pressure during the 3D printing process, and shear thickening fluids exhibit higher viscosity under pressure and tend to clog the printer nozzle. Similarly, thixotropic fluids have viscosity as a function of time, and these fluids may result in uneven distribution of particles or cells leading to inhomogeneous structures. During the printing process, the polymers are ejected from the print head through a nozzle, this causes polymers to expand after ejection. This effect is called the Barus effect. Ideal bioinks should have little or no Barus effect to preserve 3D printed object resolution [30].

The bioinks which are mostly hydrogels can be crosslinked using physical, chemical, and enzymatic methods. During the crosslinking step, the sol-gel transition occurs, and this defines the speed of printing, fidelity of the bioprinting process, and resolution. Hydrogels crosslinked using physical agents rely on non-covalent bonds for crosslinking and are generally weak. Physically-crosslinked hydrogels rely on temperature, ionic and hydrogen bonding interactions [31]. On the other hand, chemically-crosslinked hydrogels yield mechanically stable objects using 3D printing. There are many injectable hydrogels available, but for them to be used in 3D printing, they need to be fine-tuned to adjust the kinetics of crosslinking. In most of the chemically-crosslinked hydrogels, a photosensitive initiator is added to the hydrogel that forms reactive species upon exposure to ionizing radiation is used. To promote mechanical stability of printed objects, researchers have used pre- and post-fabrication crosslinking [30]. There are commercially available bioinks that offer reproducible results, such as Gel4Cell^®^, CellInk^®^, BioInk^®^, OsteoInk^®^, Bio127^®^, and BioGel^®^ [32].

## 5. Biomaterials Used in 3D Printing for Tissue Engineering

Scaffolds for TE were fabricated using 3D printing with wide range of biomaterials. These biomaterials have diversity in their chemical, mechanical, and biological properties. Scaffolds for bone TE have completely different sets of requirements compared to connective tissues. Scaffolds for bone TE needs to have mechanical properties similar to the human bone, which, again, depends on the type and location of bone. For instance, cortical bones have high compressive strengths of 100 MPa, while the spongy bones have mean compressive strengths of 3.9 MPa [33]. Apart from mechanical properties, histological properties also need to be similar. Polymers and other biomaterials are added to these scaffolds to mimic the ECM found in osseous tissue to improve cell proliferation.

In this section, we will explore composites and polymer composition used for 3D printing.

### 5.1. Ceramic and Composite Scaffolds Fabricated Using 3D Printing

Ceramics are the class of biomaterials that include metals and inorganic salts of calcium and phosphate. These biomaterials find immense potential in bone and dental TE because of their osteoconductive and osteoinductive nature. Salts of calcium and phosphate mimic the inorganic content of bone tissue. These bioresorbable biomaterials promote new bone ingrowth and are, hence, called osteoconductive. Few compounds can promote cell differentiation towards osteoblastic linage without the use of growth factors, hence, called osteoinductive. Following Table 1 summarizes novel compositions that were used in different 3D printing methods to create scaffolds for tissue engineering. Commonly-used ceramic biomaterials include β-tricalcium phosphate (β-TCP), α-tricalcium phosphate (α-TCP), hydroxy apatite (HA), bi-phasic calcium phosphate (BCP—a mixture of β-TCP and HA), calcium sulfate (CS), calcium phosphate cement (CPC), and titanium. These ceramics are often brittle in nature and are, hence, added with polymers. Biomaterials that have ceramics and polymers are categorized as composites. Commonly-added polymers include chitosan, polycaprolactone (PCL), poly lactic acid (PLA), poly l-lactide-glycolic acid (PLGA), and poly ethylene glycol diacrylate (PEGDA).

In the quest to formulate composites that have mechanical properties similar to bone, materials such as zirconium oxide, graphene, silica, and bioglass were introduced into the scaffold composition. To promote vascularization in the scaffolds, porous structures were 3D printed by many researchers. Many ceramics that are 3D printed are later subjected to sintering and freeze-drying methods to improve mechanical properties along with cytocompatibility. 3D printed scaffolds using strontium, hardystonite, gahnite, HPMC, and sodium polyacrylate was shown to have compressive strength similar to bone of 110 MPa and the scaffolds were 34% porous. These scaffolds have very large potential in bone tissue engineering because of the high mechanical properties and their ability to promote vascularization [41]. Over 500,000 bone graft procedures are done annually in the U.S. alone, with most of them using autografts [59]. Using 3D printing, patient-specific grafts can be fabricated that meet the patient needs in terms of histocompatibility, graft dimensions, and the rate of bone formation.

### 5.2. Polymer Scaffolds Fabricated Using 3D Printing

Polymers are widely-used biomaterials for 3D printing. The use of polymers in additive manufacturing is extended to many tissues, including liver, kidney and cardiac tissues, which are the most transplanted organs. Both biodegradable and non-degradable polymers are available for 3D printing, but biodegradable polymers have more advantages and are, hence, widely used. Biodegradable polymers are generally classified based on their origin as either natural or synthetic. Many synthetic polymers have been developed in recent times and have tunable degradation rates. Degradation rate is of critical importance as it needs to match the pace of new tissue formation. Combinations of natural and synthetic polymers are also used in designing the scaffold. Polymers used in bioprinting are generally in one of two physical phases, solids or liquids. Solid polymers are primarily used in FDM printers and liquid polymers are used in extrusion and inkjet printers. Liquid polymers are solutions of monomers or oligomers in a solvent system that can be polymerized or crosslinked.

Hydrogels are a type of polymer that have high capacity to hold water and, hence, mimic the environment of native tissues. They are used in a variety of applications including cell encapsulation, drug delivery systems and scaffolds [60]. Using combinations of hydrogels and cells, vascularized tissues were 3D printed with significant potential in fabricating organs. In Table 2 is a summary of recently-developed polymer compositions and strategies to develop novel scaffolds for TE.

Various 3D printing methods have been adopted in studies to create scaffolds for specific tissues. Fused deposition modelling offers an inexpensive method to create scaffolds with controlled porosity and architecture that use commercially available, biodegradable polymer filaments. However, limitations of FDM printers include thermal degradation and spatial resolution. Extrusion-based printing uses pneumatic, piston or screw driven system to create pressure, thus pushing out the suspension, solution, or emulsion. Since many hydrogels have variable viscosity, pressure-based extrusion systems prove effective in creating scaffolds. These polymers used in fabrication of scaffolds are also known as bioinks.

The liver is one of the major organs that patients wait for transplants. Recent advances in fabrication of this organ are discussed here. Using PEGDA as a hydrogel base and polydiacetylene nanoparticles were 3D-printed to create a detoxification device that proved effective against melittin toxin in murine red blood cells. These biomimetic devices were inspired from liver anatomy. The 3D printed matrix was inspired from modified liver lobule microstructures [62]. One of the challenges in fabricating liver in vitro is the inability to culture hepatocytes for many days. Using alginate-based scaffolds, hepatocytes were successfully cultured for two weeks and maintained the hepatocyte genotype. Hence, scaffolds fabricated by 3D printing holds new promise in creating functional liver tissues [66]. Using PLGA as a biomaterial, scaffolds for liver TE were fabricated and seeded with hepatocytes and non-parenchymal cells from Lewis rats. These co-cultured scaffold were incubated in static and flow conditions. The result conclude that flow conditions were more conducive for cell proliferation and viability [80].

Kidneys are the most awaited organ for transplant. Many studies have been directed in creating functional kidneys using 3D printing. Sodium alginate (5 wt %), PEGDA (40%), and calcium sulfate slurry were used to fabricate scaffolds for kidney TE. After 3D printing, these scaffolds were UV-crosslinked and seeded with human embryonic kidney cells (HEK). These scaffolds provided a conducive environment for cell viability and holds promise in kidney TE [67]. Scaffolds 3D-printed using collagen, ULGT agarose, and Fmoc dipeptide were used to seed HEK and ovine mesenchymal stem cells. These scaffolds supported high cell viability and proliferation [71]. Further research must be directed in developing bioinks for kidney and liver tissues as these organs need more transplants than any other organs.

## 6. Challenges and Future Directions

The challenges faced by tissue engineering can be seen in two categories, one category being the research and development of novel bioinks for different tissues or one universal bioink for all tissues, and other category being regulatory. Ideally, a universal bioink should be a blend of biomaterials that support native tissue viability, chemical cues, and growth factors for angiogenesis and channels for nerve innervation. These challenges can be addressed with availability of new technologies, such as additive manufacturing, that enables fabrication of complex tissues.

Vascularization is one of the most critical challenges in creating viable strategies to induce angiogenesis, including the addition of angiogenic growth factors (vascular endothelial growth factor—VEGF), the addition of platelets, bone marrow clots, and using bioreactors. Due to the ability of bioprinters to use multiple print-heads loaded with different cell types, introducing vasculature to a 3D printed construct was made possible. Kolesky et al. successfully 3D-printed thick vasculature within the tissues using multiple bioinks loaded with mesenchymal stem cells, dermal fibroblasts for extracellular matrices, and vein endothelial cells for vasculature [95].

Another approach to address the vascularization problem is to incorporate sacrificial biomaterials within the scaffold. During the 3D printing process, sacrificial materials give mechanical support while building the construct. In post-processing, these materials can be easily dissolved/removed from the constructs leaving channels or void spaces within the construct to act as vascular channels. Miller et al. have used carbohydrate glass as a sacrificial template material to fabricate a perfused vascular network within the 3D tissue [96]. Similarly, Kolesky et al. have used pluronic glass (F127) as a fugitive ink due to its excellent printability and easy removability [97].

Cui et al. have used a dual 3D printer-based system (comprised of FDM and SLA) to fabricate highly-vascularized bone constructs. Alternate layers of PLA fibers and GelMA hydrogels were deposited to build the scaffold. Growth factors, BMP2 and VEGF, were used for osteogenesis and angiogenesis, respectively, and the constructs were subjected to perfusion culture in a bioreactor to form an intricate vascular bone construct [9].

Another category of challenge is translation of this research to next level, i.e., making these advancements in RM available to patients. This area is more challenging because of the regulatory factors that need to be cleared. The Food and Drug Administration (FDA) has regulations, such as 510 K, and many other requirements that need to be met before reaching the patient. Currently, the 3D-printed tissues and scaffolds are used for screening purposes and evaluation in animal models. 

## Figures and Tables

**Table 1:** Ceramic and composite scaffolds fabricated using 3D printing for use in tissue engineering.

Ceramics Composition	Polymer	3D Printing Method	Reference
BCP, HPMC, ZrO_2_		Pressure extrusion	[34]
Mesoporous bioglass, CS		Pressure extrusion	[35]
CS		Inkjet	[36]
Mesoporous silica, CPC		Pressure extrusion	[37]
Wallastonite, magnesium		Pressure extrusion	[38]
Tricalcium phosphate, phosphoric acid		Inkjet	[39]
Silica, calcium carbonate		Laser assisted gelling	[40]
Strontium, hardystonite, gahnite, HPMC	Sodium polyacrylate	Extrusion	[41]
CPC (Osteoflux)		Pressure extrusion	[42]
Ti6Al4V		Laser beam melting	[43]
Calcium chloride, glutamic acid, ammonium hydrogen phosphate	Sodium alginate	Pressure extrusion	[44]
HA, α-TCP, phosphoric acid	Collagen	Inkjet	[45]
Titanium, platelets	Gelatin	Laser sintering	[46]
HA, solvent system	PLGA	Extrusion	[47]
Calcium silicate, magnesium	PCL	Laser sintering	[48]
HA, PLGA microspheres	PCL	FDM	[49]
Graphene	PCL	FDM	[50]
HA, bone marrow clots	PCL	FDM	[51]
HA	PCL	FDM	[52]
BCP	PLGA, PCL, collagen	FDM	[53]
BCP	PCL	Inkjet	[54]
β-TCP	PCL	FDM	[55]
β-TCP	PEGDA	Stereolithiography	[56]
HA	PLA	FDM	[57,58]

**Table 2:** Polymer scaffolds fabricated using 3D printing for use in tissue engineering.

Scaffold Composition	3D Printing Method	Target Tissue	Reference
Pluronics, gelatin methacrylate	Pressure extrusion	Vascular	[61]
PEGDA, polydiacetylene nanoparticles	Stereolithography	Liver	[62]
PCL, chitosan	FDM	Bone	[63]
PCL, castor oil	FDM	Bone	[64]
Vinylester, vinylcarbonate	DLP	Bone	[65]
Alginate	Pressure extrusion	Liver	[66]
Alginate, PEGDA, CS	Extrusion	Kidney	[67]
Alginate	Extrusion	Microphysiologic studies	[68]
Alginate, gelatin	Extrusion	Mutlicellular tissue	[69]
Gelatin methacrylate, alginate, poly ethylene glycol tetra acrylate	Extrusion	Vascular	[70]
Agarose, collagen	Extrusion	Kidney	[71]
Gelatin	Extrusion	Ovary	[11]
Cellulose nanocrystal	DIW	Multicellular tissue	[72]
Nanofibrillated cellulose (NFC), alginate	Pressure extrusion	Cartilage	[73]
Collagen, chitosan	Extrusion	Neural	[74]
Alginate, gelatin	Extrusion	Tumor microenvironment	[75]
Alginate, collagen, agarose	Extrusion	Cartilage	[76]
Collagen	Pressure extrusion	Skin	[12]
PVA, phytagel	Extrusion	Soft connective tissue	[77]
Gelatin, silk fibroin	Extrusion	Skin	[78]
Hyaluronic acide, gelatin	Extrusion	Cardiac	[79]
PLGA	Inkjet	Liver	[80]
Matrigel, agarose	Extrusion	Intestinal	[81]
Methacrylated hyaluronic acid (Me-HA), metharylated gelatin	Extrusion	Cardica valve	[82]
Me-HA	Extrusion	Bone	[83]
Agarose, single wall carbon nanotubes	Extrusion	Biosensors, various tissues	[84]
NFC, alginate, hyaluronic acid	Pressure extrusion	Cartilage	[85]
Nanocrystalline HA, PLGA	Stereolithography	Bone	[86]
Poly (l-lactide-*co*-ε-caprolactone)	FDM	Tubular, muscle	[87]
PCL	FDM	Bone	[88]
PCL, PLGA, collagen, gelatin	FDM, extrusion	Bone	[89]
PLA, PLGA, collagen	FDM	Tendon-bone	[90]
PLA, collagen	FDM	Bone	[91]
PLA	FDM	Osteochondral	[92]
PLA, acrylonitrile butadiene styrene	FDM	Osteochondral	[93]
PLA	FDM	Bone	[94]
